# Common risk factors and edentulism in adults, aged 50 years and over, in China, Ghana, India and South Africa: results from the WHO Study on global AGEing and adult health (SAGE)

**DOI:** 10.1186/s12903-016-0256-2

**Published:** 2016-07-27

**Authors:** Alexander Kailembo, Raman Preet, Jennifer Stewart Williams

**Affiliations:** 1 Elite Dental Clinic, 6th Floor, Oasis Office Complex, Plot No. 14/15, Haile Selassie Road, P.O Box 38394, Masaki, Dar es salaam Tanzania; 2 Unit of Epidemiology and Global Health, Department of Public Health and Clinical Medicine, Faculty of Medicine, Umeå University, Umeå, Sweden; 3 Research Centre for Generational Health and Ageing, Faculty of Health, University of Newcastle, Newcastle, Australia

**Keywords:** Oral health, Tooth loss, Periodontal, Caries, Common risk factor approach, CRFA, Non-communicable diseases, NCDs, Low- and middle-income countries, LMICs

## Abstract

**Background:**

Edentulism (loss of all teeth) is a final marker of disease burden for oral health common among older adults and poorer populations. Yet most evidence is from high-income countries. Oral health has many of the same social and behavioural risk factors as other non-communicable diseases (NCDs) which are increasing rapidly in low- and middle-income countries with ageing populations. The “common risk factor approach” (CRFA) for oral health addresses risk factors shared with NCDs within the broader social and economic environment.

**Methods:**

The aim is to improve understanding of edentulism prevalence, and association between common risk factors and edentulism in adults aged 50 years and above using nationally representative samples from China (*N* = 11,692), Ghana (*N* = 4093), India (*N* = 6409) and South Africa (*N* = 2985). The data source is the World Health Organization (WHO) Study on global AGEing and adult health (SAGE) Wave 1 (2007–2010). Multivariable logistic regression describes association between edentulism and common risk factors reported in the literature.

**Results:**

Prevalence of edentulism: in China 8.9 %, Ghana 2.9 %, India 15.3 %, and South Africa 8.7 %. Multivariable analysis: in China, rural residents were more likely to be edentulous (OR 1.36; 95 % CI 1.09–1.69) but less likely to be edentulous in Ghana (OR 0.53; 95 % CI 0.31–0.91) and South Africa (OR 0.52; 95 % CI 0.30–0.90). Respondents with university education (OR 0.31; 95 % CI 0.18–0.53) and in the highest wealth quintile (OR 0.68; 95 % CI 0.52–0.90) in China were less likely to be edentulous. In South Africa respondents with secondary education were more likely to be edentulous (OR 2.82; 95 % CI 1.52–5.21) as were those in the highest wealth quintile (OR 2.78; 95 % CI 1.16–6.70). Edentulism was associated with former smokers in China (OR 1.57; 95 % CI 1.10–2.25) non-drinkers in India (OR 1.65; 95 % CI 1.11–2.46), angina in Ghana (OR 2.86; 95 % CI 1.19–6.84) and hypertension in South Africa (OR 2.75; 95 % CI 1.72–4.38). Edentulism was less likely in respondents with adequate nutrition in China (OR 0.68; 95 % CI 0.53–0.87). Adjusting for all other factors, compared with China, respondents in India were 50 % more likely to be edentulous.

**Conclusions:**

Strengthening the CRFA should include addressing common determinants of health to reduce health inequalities and improve both oral and overall health.

## Background

Oral health is fundamental for general health, functioning and well-being. The Global Burden of Disease 2010 Study estimated that oral conditions (untreated caries, severe periodontitis and severe tooth loss) accounted for almost 2 % of all years lived with disability. Disability adjusted life-years (DALYs) due to oral conditions increased by 20.8 % between 1990 and 2010, largely due to population growth and ageing [1–3]. This epidemiological study describes patterns of edentulism (loss of all teeth) in four low- and middle-income countries (LMICs) in Asia and Africa. Although DALYs for edentulism have fallen world-wide, the profile of edentulism is not homogeneous between or within countries and evidence of oral health in LMICs is limited [4].

Scientific and technological developments in oral health treatments and improved hygiene have helped prevent and control diseases of the mouth, yet advances in oral health science have largely benefited more advantaged populations in high-income countries (HICs). Research aimed at improving oral health should take into account social as well as biological determinants. People are now living longer in all parts of the world and the impact of poor oral health on the quality of life of older adults is an important public health issue [5]. In particular, efforts need to be strengthened in LMICs [3, 6–8] where periodontal diseases and caries are often “solved” by tooth/teeth extraction instead of conservation [9–12].

Observational evidence points to tobacco smoking [13, 14], alcohol consumption [15, 16] and poor nutrition [9, 17] as behavioural risk factors for edentulism. Studies also show that socio-demographic characteristics such as increasing age, gender, rural geo-locality, less education, and lower socioeconomic status [18–24] are also predictors of edentulism. In addition, both clinical and epidemiological associations have been observed between periodontal diseases and other chronic non-communicable diseases (NCDs) such as type 2 diabetes [25], angina pectoris [26], hypertension [27] and respiratory [28] and cardiovascular diseases [6, 29]. However all of these relationships vary according to the characteristics of the populations being studied [29].

In 2015, the proportion of people in the world aged 65 and above was 8.5 % of the total 7.3 billion people worldwide. This segment of the global population is expected to increase by more than 60 %, from 617.1 million to about one billion, between 2015 and 2030 - equivalent to about 12 % of the world’s population. The proportion of older people will continue to grow in the following 20 years. By 2050 people in the world aged 65 and above will comprise about 16.7 % of the estimated total population of 9.4 billion people. Therefore the average annual increase in the sheer numbers of people aged 65 and above between 2015 and 2050 will be 27.1 million [30].

Although the proportion of people aged 65 and above within the Asian region was 7.9 % in 2015 this translates to 341.1 million people or 5.3 % of the people in the world aged 65 and above. By 2050, about 975.3 million, or two thirds of the world’s population of people aged 65 and above will live in the Asian region. These figures are dominated by China and India, both of which have current populations exceeding one billion. In contrast, the African region has a relatively young age structure with about 3.5 % of the population aged 65 years and above in 2015. Yet the Region is facing major demographic shifts. It is estimated that by 2050 this proportion of older adults in the Region will double to 6.7 % being an almost fourfold increase from 40.6 million to 150.5 million adults aged 65 years and above between 2015 and 2050 [30, 31].

As the sheer numbers of older adults increases over the next decade, so will the burden of chronic periodontal and other NCDs, with a disproportionately higher share expected in LMICs [32]. Over the next 20 years the overall burden of NCDs is predicted to increase from 60 to 79 % in Asia and from 28 to 51 % in Africa [33].

Oral health has many of the same social and behavioural risk factors as other NCDs and oral health promotion is being given increasing attention in developed countries [34]. The “common risk factor approach” (CRFA) addresses risk factors shared with NCDs within the broader social and economic environment. Tobacco use, for example, is linked to periodontal disease, tooth loss, and 90 % of all lung cancers. Unhealthy diets are associated with coronary heart disease, type 2 diabetes, strokes, many cancers and dental caries. The CRFA offers an efficacious method of health promotion and illness prevention, which highlights the importance of the mouth-body connection for overall health and well-being [35].

 The CRFA aims to address the broader social and economic determinants (of health) which, in turn, can help reduce unfair health inequalities and inequities in health [36, 37]. Some argue that failure to reduce inequalities in health in many HICs has been because strategies have focused too much on lifestyle change at the expense of not directing efforts and resources towards the broader social determinants of health [36, 38]. Inequalities in oral health reflect the same socioeconomic gradient that occurs in all populations whereby the poor and disadvantaged have the worst health [36, 37, 39, 40].

Edentulism is a “final marker of disease burden for oral health” [12, 41] and an important indicator of dental caries [42–44]. If governments in developing countries are to adopt the CRFA to address oral health they need reliable standardised nationally representative epidemiological data and processes by which to convert evidence into information for policy and planning [7]. However evidence on oral health from developing countries is not consistent.

The oral health needs of older people in the African continent [45] are significant with edentulism prevalent in the poorest communities, among older adults and among the less educated [10]. Reports of edentulism prevalence in Asia are varied. In a study of community-dwelling adults aged 60 and above in India, almost one in six were completely edentulous and over 60 % of dentate subjects had decayed teeth or root caries [46]. Yet a study of community-dwelling 65 to 74 year olds in China found that the prevalence of edentulism was only 4.5 % and 3.5 % in urban and rural residents respectively [47]. There is a clear need to ensure comparability between sampling and methods of data collection in epidemiological studies in developing countries where oral health is an emerging public health issue [48, 49].

A recent epidemiological study by Peltzer et al. [50] investigated factors associated with edentulism in a pooled six-country data set comprising China, Ghana, India, Mexico, Russia and South Africa. The authors identified older age, lower education, NCDs, tobacco use, and inadequate fruit and vegetable consumption as risk factors for edentulism. However, the pooled analysis masks important differences within and between countries. In this study, we use a similar nationally comparable standardised study sample of adults to specifically investigate common risk factors for edentulism at the country level. The aim is to improve understanding of edentulism prevalence and association between common risk factors and edentulism (self-reported) in adults, aged 50 years and above, in China, Ghana, India and South Africa.

## Methods

### Data collection

The data source for this study is the World Health Organization (WHO) Study on global AGEing and adult health (SAGE) Wave 1 (2007–2010) which aims to address the gap in reliable and scientific knowledge on ageing and adult health in LMICs. WHO-SAGE is a longitudinal study of nationally representative samples of adults aged 50 and above in China, Ghana, India, Mexico, Russia and South Africa. Smaller non-representative samples of adults aged 18 to 49 years were collected for comparative purposes. Data analysed here were collected from face-to-face interviews using structured household and individual questionnaires.

WHO-SAGE employed a stratified random sampling strategy in all countries, with households as the final sampling units. The strata ensure representation of a range of living conditions and urban and rural localities in each country. Household-level analysis weights and person-level analysis weights were calculated for each country with post-stratification weights to adjust for age and sex distributions and non-response [51]. When multiple countries are analysed as a single data set, age and sex standardisations based on WHO’s World Standard Population [52] adjust for between-country age and sex differences. WHO-SAGE data sets, including sampling weights, are in the public domain. Further details of WHO-SAGE are given elsewhere [53].

### Study variables

The binary dependent variable “edentulism” was derived from answers to the question in the individual questionnaire: “Have you lost all your natural teeth?” Those who responded “yes” were defined as edentulous, and those who responded “no” to this question were categorized as being “dentate”.

The choice of independent variables, or “common risk factors” was informed by the literature [35, 36]. Socio-demographic variables are sex, age, residence, education and wealth status. Sex is male or female. The age categories are 50–59 years vs. 60–69 years vs. 70–79 years vs. 80+ years. Residence (geo-locality) is urban or rural. Education refers to the highest reported achieved level of education and is grouped no schooling vs. primary vs. secondary vs. university or college. All of these independent variables were directly derived from the individual questionnaires.

Information on ownership of household assets was taken directly from household questionnaires and converted to a household wealth status variable. This was then linked to the individual questionnaires. The SAGE household questionnaire captures information on household characteristics (eg cooking oil, floor and roof types), ownership of durable goods (eg radio, car) and access to basic services (eg electricity, clean water and sanitation). In order to construct an asset-based index from this information it was necessary to develop weights to assign to household assets. Principal Components Analysis was used to generate weights from which raw continuous scores, indicative of household wealth, were derived. These scores were transformed into “wealth quintiles” with quintile one representing the lowest wealth and quintile five the highest [54, 55]. The above steps were undertaken by WHO.

Health behaviours are smoking (non-smoker vs. daily smoker vs. non-daily smoker vs. former smoker) alcohol use (drinkers vs. non-drinkers) and nutritional status derived from answers to the questions; “How many servings of fruit do you eat on a typical day?” and “How many servings of vegetables do you eat on a typical day?” Respondents with five or more servings are “adequate” and those with less than five servings are “inadequate”.

Chronic conditions are diabetes, angina and hypertension. Binary variables denote yes or no answers to questions that asked whether the respondents had “ever been diagnosed” with the condition. A country dummy variable (with China as the reference group) is included in the pooled multivariable analysis to adjust for country differences.

### Study sample

The available data set of WHO SAGE Wave 1 individual respondents from China, Ghana, India, Mexico, Russia and South Africa comprised 47,443 respondents. The data for Mexico (*n* = 5548) and Russia (*n* = 4947) were not included because of high percentages of missing data on the dependent variable (52 % in Mexico and 13 % in Russia).

The analysis in this study compares two sets of countries in two distinct geographic regions – China and India in Asia and Ghana and South Africa in Sub-Saharan Africa. Individuals who did not complete the SAGE individual questionnaire were not included – China (*n* = 237), Ghana (*n* = 463), India (*n* = 968) and South Africa (*n* = 2). Respondents aged less than 50 years were also excluded from the study – China (*n* = 1636), Ghana (*n* = 805), India (*n* = 4670) and South Africa (*n* = 385). The potential study sample comprised SAGE Wave 1 respondents in China (*n* = 13,177), Ghana (*n* = 4305), India (*n* = 6560) and South Africa (*n* = 3840) who completed the surveys and were aged 50 years and over (*N* = 27,882). The final country study samples are reported in the Results section.

### Statistical analysis

Only records with complete data on all study variables were analysed. Individual country samples are described by socio-demographic factors, health behaviours and chronic conditions. The prevalence of edentulism is estimated with 95 % confidence intervals (CIs) to allow statistical comparisons between countries. Chi-squared tests show differences in prevalence by socio-demographic characteristics, behaviours and chronic conditions.

Univariable logistic regressions describe associations between socio-demographic factors, health behaviours and chronic conditions and edentulism in each country, and in the pooled data set. The criterion for inclusion in the multivariable analyses was set at *p* < 0.05 in the pooled analysis. In some cases, eg sex in Ghana, India and South Africa, associations were not significant in the univariate analyses, but the variables were retained because of evidence of association with edentulism in the literature. Associations are presented as odds ratios (ORs) and 95 % CIs.

All analyses applied either country or pooled weights appropriate for making estimates representative of the populations. The analyses were carried out using STATA 13 software (StataCorp, 2013).

## Results

The study sample of adults aged 50 years and over in the four countries pooled was 25,179. The largest sample was China (*n* = 11,692), followed by India (*n* = 6409), Ghana (*n* = 4093) and South Africa (*n* = 2985). Proportions of females to males were just above 50 % in China and South Africa and just under 50 % in Ghana and India. Overall almost half the study sample was aged 50–59 years, about 5 % were aged 80+ years, and 58 % were rural residents (Table 1).

**Table 1:** Weighted characteristics of the study population of adults aged 50+ years in China, Ghana, India and South Africa and pooled, SAGE Wave 1 (*N* = 25,179)

Characteristics	China		Ghana		India		South Africa		Pooled data	
	*N* = 11,692		*N* = 4093		*N* = 6409		*N* = 2985		*N* = 25,179	
	*n*	(%)	*n*	(%)	*n*	(%)	*n*	(%)	*n*	(%)
Sex										
Male	5492	49.7	2127	52.0	3230	50.6	1189	49.8	12,038	49.6
Female	6200	50.3	1966	48.0	3179	49.4	1796	50.2	13,141	50.4
Age group (years)										
50–59	5067	44.9	1619	40.2	2862	47.9	1313	49.5	10,861	49.8
60–69	3555	32.3	1136	27.2	2182	31.2	965	30.9	7838	29.4
70–79	2464	18.6	931	22.9	1044	16.3	517	14.1	4956	15.9
80+	606	4.2	407	9.7	321	4.6	190	5.5	1524	4.9
Residence										
Urban	6030	49.2	1659	40.6	1654	29.0	2006	65.3	11,349	41.6
Rural	5662	50.8	2434	59.4	4755	71.0	979	34.7	13,830	58.4
Highest education										
No schooling	2727	21.7	2257	53.9	3286	51.7	771	24.1	9041	31.2
Primary or less	4384	40.0	855	21.4	1642	25.0	1444	46.6	8325	25.2
Secondary	4014	33.6	837	21.0	1162	18.3	617	23.3	6630	29.0
University	567	4.7	144	3.7	319	5.0	153	6.0	1183	4.6
Wealth quintile										
1 (Lowest)	2259	15.7	812	18.2	1043	18.2	548	19.4	4662	16.5
2	2284	17.8	802	19.0	1185	19.2	608	20.2	4879	18.3
3	2337	20.3	828	20.9	1176	18.7	608	19.4	4949	19.6
4	2429	23.6	843	20.9	1393	20.0	612	20.0	5277	22.4
5 (Highest)	2383	22.6	808	21.0	1612	23.9	609	21.0	5412	23.2
Smoking										
Non-smoker	7794	64.1	3037	75.2	3048	45.7	1934	66.9	15,813	58.5
Daily smoker	2849	26.7	402	8.1	2826	46.5	664	20.1	6741	32.8
Non-daily smoker	302	2.5	112	2.6	195	3.0	115	3.2	724	2.7
Former smoker	747	6.7	542	14.1	340	4.8	272	9.8	1901	6.0
Alcohol use										
Drinker	3612	33.9	2389	57.8	1015	15.9	827	25.4	7843	28.3
Non-drinker	8080	66.1	1704	42.2	5394	84.1	2158	74.6	17,336	71.7
Nutritional status										
Inadequate	1413	9.6	2840	68.0	5711	90.4	2186	68.4	12,150	35.9
Adequate	10,279	90.4	1253	32.0	698	9.6	799	31.6	13,029	64.1
Chronic conditions										
Diabetes										
Yes	785	6.7	161	3.9	471	6.5	297	9.6	1714	6.3
No	10,907	93.3	3932	96.1	5938	93.5	2688	90.6	23,465	93.7
Angina										
Yes	1059	8.0	138	3.7	320	5.5	176	4.9	1693	6.8
No	10,633	92.0	3955	96.3	6084	94.5	2809	95.1	23,486	93.2
Hypertension										
Yes	3254	26.8	548	14.0	1120	16.8	922	31.0	5844	22.8
No	8438	73.2	3545	86.0	5289	83.2	2063	69.0	19,335	77.2

Table 2 gives the prevalence of edentulism by country and sample characteristics. Overall in the four countries, prevalence was 10.9 % (*N* = 2591). Prevalence was highest in India at (15.3 %; 95 % CI 13.4–17.5) and lowest in Ghana (2.9 %; 95 % CI 2.3–3.6). Prevalence was significantly different between countries except for China (8.9 %; 95 % CI 8.1–9.8) and South Africa (8.7 %; 95 % CI 7.0–10.8). In all four countries, the prevalence of edentulism was higher in females but the sex difference was significant (*p* < 0.001) only in China (44.4 % male vs. 55.6 % female). The prevalence of edentulism was significantly different (*p* < 0.001) by age in China, Ghana and India. Edentulism was more prevalent in rural areas in China (57.9 %) and in urban areas in Ghana (59.3 %) and South Africa (83.7 %). These differences were significant in China (*p* = 0.005), Ghana (*p* = 0.020) and South Africa (*p* < 0.001). Differences in education gradients were observed in China (*p* < 0.001) and South Africa (*p* = 0.004). The wealth gradient was also significant in China and South Africa (*p* < 0.001 for both). The country comparison for smoking status was significant for China (*p* < 0.001) and South Africa (*p* = 0.020) and for alcohol use the differences were significant for China (*p* = 0.001) and India (*p* = 0.001). There was a significant difference in nutritional status in China only (*p* < 0.001). The prevalence of diabetes was significantly different in Ghana (*p* = 0.012) and South Africa (*p* = 0.001), the prevalence of angina was significantly different in China (*p* = 0.001) and Ghana (*p* = 0.011), and the prevalence of hypertension was significantly different in China and South Africa (*p* < 0.001).

**Table 2:** Weighted prevalence of self-reported edentulism according to characteristics of study population of adults aged 50+ years in China, Ghana, India and South Africa and pooled, SAGE Wave 1

Characteristics	China			Ghana			India			South Africa			Pooled data		
	*N*	%	95 % CI	*N*	%	95 % CI	*N*	%	95 % CI	*N*	%	95 % CI	*N*	%	95 % CI
Prevalence	1250	8.9	8.1–9.8	112	2.9	2.3–3.6	916	15.3	13.4–17.5	313	8.7	7.0–10.8	2591	10.9	9.9–11.9
Sex	*n*	%	*P*-value	*n*	%	*P*-value	*n*	%	*P*-value	*n*	%	*P*-value	*n*	%	*P*-value
Male	555	44.4	<0.001	47	45.0	0.220	436	47.0	0.274	105	38.2	0.713	1143	44.7	0.004
Female	695	55.6		65	55.0		480	53.0		208	61.8		1448	55.3	
Age group (years)															
50–59	161	13.9	<0.001	27	24.9	<0.001	225	30.8	<0.001	105	36.5	0.072	518	22.0	<0.001
60–69	335	30.0		24	20.5		296	29.4		104	34.2		759	28.4	
70–79	526	39.2		25	24.5		278	31.1		79	20.0		908	34.2	
80+	228	16.9		36	30.1		117	8.7		25	9.3		406	15.4	
Residence															
Urban	540	42.1	0.005	58	59.3	0.020	245	35.4	0.074	277	83.7	<0.001	1120	36.2	0.036
Rural	710	57.9		54	45.7		671	64.6		36	16.3		1471	63.8	
Highest education															
No schooling	531	42.0	<0.001	74	62.2	0.551	499	55.5	0.100	36	10.0	0.004	1140	49.4	<0.001
Primary or less	464	39.5		17	17.9		259	25.8		176	51.5		916	33.1	
Secondary	224	16.8		17	15.7		127	14.9		93	34.4		461	15.2	
University	31	1.7		4	4.2		31	3.8		8	4.1		74	2.4	
Wealth quintile															
1 (Lowest)	386	26.6	<0.001	24	17.8	0.861	136	18.8	0.854	28	8.5	<0.001	574	23.7	<0.001
2	274	21.4		21	16.8		176	18.1		48	14.8		519	19.9	
3	264	22.9		24	24.7		178	17.5		74	16.5		540	20.0	
4	197	16.7		20	17.8		195	21.4		75	21.4		487	18.4	
5 (Highest)	129	12.4		23	23.0		231	24.2		88	38.8		471	18.0	
Smoking															
Non-smoker	842	64.6	<0.001	85	72.6	0.300	429	48.1	0.150	187	57.6	0.020	1543	57.2	0.020
Daily smoker	273	23.7		9	6.7		385	42.2		94	29.6		761	32.3	
Non-daily smoker	22	1.5		1	0.6		31	3.5		5	0.4		59	2.4	
Former smoker	113	10.2		17	20.1		71	6.2		27	12.4		228	8.1	
Alcohol use															
Drinker	329	28.8	0.001	58	53.3	0.317	116	11.0	0.001	79	22.9	0.576	582	20.4	<0.001
Non-drinker	921	71.2		54	46.7		800	89.0		234	77.1		2009	79.6	
Nutritional status															
Inadequate	253	16.7	<0.001	75	66.9	0.816	798	89.4	0.500	235	74.2	0.207	1361	51.7	<0.001
Adequate	997	83.3		37	33.1		118	10.6		78	25.8		1230	48.3	
Chronic conditions															
Diabetes															
Yes	101	7.8	0.210	8	9.0	0.012	77	8.4	0.135	68	18.0	0.001	254	7.7	0.067
No	1149	92.2		104	91.0		839	91.6		245	82.0		2337	92.3	
Angina															
Yes	173	11.8	0.001	7	10.1	0.011	55	5.7	0.827	33	7.2	0.146	268	8.6	0.018
No	1077	88.2		105	89.9		861	94.3		280	92.8		2323	91.4	
Hypertension															
Yes	465	34.5	<0.001	10	11.8	0.603	175	16.3	0.777	173	57.7	<0.001	823	25.5	0.026
No	785	65.5		102	88.2		741	83.7		140	42.3		1768	74.5	

Table 3 reports the results of univariable logistic regressions of common risk factors associated with edentulism. Women had about 30 % higher odds of being edentulous in China (*p* < 0.001). Age was significantly associated with edentulism in China and India whereby older aged respondents were more likely to be edentulous (*p* < 0.001). Rural residents in China were significantly (*p* = 0.005) more likely to be edentulous, and urban residents in Ghana (*p* = 0.020) and South Africa (*p* < 0.001) were more likely to be edentulous. An education gradient was evident in China wherby the more educated groups were significantly less likely to be edentulous (*p* < 0.001). The pattern was reversed in South Africa where respondents who had primary or secondary schooling were significantly more likely to be edentulous compared with those who had no schooling (*p* < 0.001). There were also country differences in the direction of association with smoking. Compared with non-smokers, the odds of former smokers being edentulous were about 60 % higher in China (*p* < 0.001) and the odds of daily smokers being edentulous were about 80 % higher in South Africa (*p* = 0.033). Compared with drinkers, the odds of non-drinkers being edentulous were 30 % higher in China and 60 % higher in India (*p* < 0.001 both). In China respondents with adequate nutritional status were half as likely to be edentulous (*p* < 0.001), respondents with angina had 60 % higher odds of being edentulous (*p* = 0.001) and the odds of respondents with hypertension being edentulous were about 50 % higher (*p* < 0.001). In Ghana respondents with angina were three times more likely to be edentulous (*p* = 0.016) and respondents with diabetes were over two and a half times more likely to be edentulous (*p* = 0.015). In South Africa the odds of respondents with diabetes being edentulous were 2.3 times higher (*p* = 0.002) and the odds of respondents with hypertension being edentulous were about 3.4 times higher (*p* < 0.001).

**Table 3:** Univariable logistic regression of common risk factors associated with edentulism, adults aged 50+ years in China, Ghana, India and South Africa and pooled, SAGE Wave 1 (weighted)

Characteristics	China		Ghana		India		South Africa		Pooled data	
	OR (95 % CI)	*p*-value	OR (95 % CI)	*p*-value	OR (95 % CI)	*p*-value	OR (95 % CI)	*p*-value	OR (95 % CI)	*p*-value
Sex										
Male	1		1		1		1		1	
Female	1.27(1.14–1.40)	<0.001	1.33(0.84–2.12)	0.221	1.19(0.87–1.63)	0.274	1.09(0.70–1.68)	0.713	1.25(1.07–1.45)	0.004
Age group (years)										
50–59	1		1		1		1		1	
60–69	3.19(2.52–4.04)	<0.001	1.22(0.64–2.32)	0.537	1.55(1.24–1.95)	<0.001	1.55(0.91–2.65)	0.105	2.32(1.98–2.71)	<0.001
70–79	8.16(6.14–10.85)	<0.001	1.75(0.85–3.61)	0.128	3.80(2.81–5.13)	<0.001	2.05(1.23–3.41)	0.006	6.02(4.95–7.33)	<0.001
80+	20.09 (14.31–28.20)	<0.001	5.45(3.05–9.75)	0.000	3.79(2.62–5.49)	<0.001	2.60(0.86–7.66)	0.093	10.19(7.86–13.23)	<0.001
Residence										
Urban	1		1		1		1		1	
Rural	1.36(1.10–1.69)	0.005	0.57(0.35–0.91)	0.020	0.71(0.48–1.04)	0.075	0.34(0.19–0.59)	<0.001	1.30(1.02–1.65)	0.036
Highest education										
No schooling	1		1		1		1		1	
Primary	0.46(0.39–0.55)	<0.001	0.72(0.31–1.64)	0.429	0.9(0.7–1.2)	0.669	2.81(1.64–4.81)	<0.001	0.55(0.47–0.63)	<0.001
Secondary	0.22(0.17–0.29)	<0.001	0.64(0.35–1.17)	0.143	0.7(0.5–0.9)	0.041	3.90(2.04–7.48)	<0.001	0.29(0.24–0.35)	<0.001
University	0.15(0.09–0.27)	<0.001	1.00(0.30–3.32)	0.996	0.7(0.4–1.1)	0.115	1.64(0.48–5.56)	0.426	0.29(0.20–0.44)	<0.001
Wealth quintile										
1 (lowest)	1		1		1		1		1	
2	0.67(0.53–0.85)	0.001	0.90(0.44–1.83)	0.762	0.95(0.74–1.23)	0.549	1.72(0.66–4.46)	0.263	0.73(0.60–0.88)	0.001
3	0.62(0.53–0.73)	<0.001	1.21(0.55–2.65)	0.634	0.90(0.65–1.26)	0.504	2.02(0.86–4.77)	0.107	0.68(0.57–0.80)	<0.001
4	0.38(0.30–0.48)	<0.001	0.86(0.43–1.75)	0.680	1.05(0.72–1.51)	0.810	2.61(1.15–5.92)	0.022	0.53(0.42–0.67)	<0.001
5 (highest)	0.29(0.21–0.39)	<0.001	1.11(0.55–2.24)	0.761	0.98(0.69–1.37)	0.890	4.85(2.08–11.33)	<0.001	0.50(0.40–0.63)	<0.001
Smoking										
Non-smoker	1		1		1		1		1	
Daily smoker	0.87(0.71–1.07)	0.172	0.85(0.43–1.68)	0.635	0.84(0.66–1.08)	0.171	1.81(1.05–3.13)	0.033	1.01(0.86–1.18)	0.908
Non-daily smoker	0.57(0.36–0.91)	0.019	0.23(0.03–1.70)	0.149	1.13(0.65–1.96)	0.660	0.14(0.03–0.70)	0.017	0.90(0.62–1.33)	0.604
Former smoker	1.59(1.24–2.04)	<0.001	1.50(0.68–3.30)	0.314	1.30(0.86–1.94)	0.209	1.53(0.79–2.96)	0.207	1.45(1.20–1.78)	<0.001
Alcohol use										
Drinker	1		1		1		1		1	
Non-drinker	1.30(1.12–1.50)	<0.001	1.21(0.83–1.75)	0.318	1.64(1.22–2.20)	<0.001	1.16(0.68–2.00)	0.577	1.61(1.40–1.85)	<0.001
Nutritional status										
Inadequate	1		1		1		1		1	
Adequate	0.49(0.39–0.62)	<0.001	1.06(0.66–1.69)	0.816	1.15(0.77–1.70)	0.499	0.73(0.45–1.19)	0.208	0.48(0.40–0.58)	<0.001
Chronic Conditions										
Angina	1.60(1.22–2.12)	0.001	3.13(1.24–7.93)	0.016	1.06(0.65–1.72)	0.827	1.58(0.85–2.94)	0.149	1.3(31.05–1.70)	0.019
Diabetes	1.20(0.90–1.60)	0.210	2.56(1.20–5.46)	0.015	1.38(0.90–2.13)	0.137	2.28(1.36–3.82)	0.002	1.27(0.98–1.65)	0.067
Hypertension	1.49(1.27–1.76)	<0.001	0.82(0.38–1.75)	0.604	0.96(0.71–1.29)	0.777	3.43(2.18–5.41)	<0.001	1.19(1.02–1.38)	0.026

In multivariable logistic regression (Table 4) edentulousness was significantly associated with the oldest respondents (aged 80+) and females in Ghana (OR 1.56; 95 % CI 1.01–2.42), holding all other variables constant. In China association between female sex and edentulism attenuated to non-significance due to confounding, yet age remained significant in the presence of sex and all other covariates. Rural residents in China were significantly more likely to be edentulous (OR 1.36; 95 % CI 1.109–1.69) but rural residents were less likely to be edentulous in Ghana (OR 0.53; 95 % CI 0.31–0.91) and South Africa (OR 0.52; 95 % CI 0.30–0.90) when holding all other variables constant.

**Table 4:** Multivariable logistic regression of common risk factors associated with edentulism, adults aged 50+ years in China, Ghana, India and South Africa and pooled, SAGE Wave 1 (weighted)

China			Ghana		India		South Africa		Pooled data	
Variable	OR (95 % CI)	*p*-value	OR (95 % CI)	*p*-value	OR (95 % CI)	*p*-value	OR (95 % CI)	*p*-value	OR (95 % CI)	*p*-value
Sex										
Male	1		1		1		1		1	
Female	1.09 (0.86–1.37)	0.477	1.56 (1.01–2.42)	0.044	1.01 (0.67–1.52)	0.959	1.07 (0.66–1.74)	0.789	0.96 (0.77–1.20)	0.716
Age group										
50–59	1		1		1		1		1	
60–69	2.80 (2.25–3.50)	<0.001	1.16 (0.62–2.20)	0.635	1.51 (1.20–1.92)	0.001	1.45 (0.86–2.43)	0.162	2.03 (1.74–2.36)	<0.001
70–79	6.74 (5.22–8.70)	<0.001	1.73 (0.83–3.59)	0.140	3.80 (2.84–5.09)	<0.001	1.70 (0.98–2.96)	0.059	4.94 (4.10–5.96)	<0.001
80+	14.89 (10.88–20.37)	<0.001	5.25 (2.80–9.83)	<0.001	3.59 (2.50–5.16)	<0.001	2.89 (1.29–6.45)	0.010	7.67 (5.93–9.91)	<0.001
Residence										
Urban	1		1		1		1		1	
Rural	1.36 (1.09–1.69)	0.007	0.53 (0.31–0.91)	0.022	0.70 (0.45–1.08)	0.110	0.52 (0.30–0.90)	0.021	1.01 (0.77–1.32)	0.964
Education										
No schooling	1		1		1		1		1	
Primary	0.82 (0.67–1.00)	0.048	1.05 (0.44–2.53)	0.915	0.94 (0.72–1.22)	0.637	2.32 (1.31–4.12)	0.004	0.88 (0.75–1.03)	0.105
Secondary	0.57 (0.44–0.75)	<0.001	0.98 (0.51–1.90)	0.960	0.76 (0.56–1.03)	0.081	2.82 (1.52–5.21)	0.001	0.58 (0.48–0.71)	<0.001
University	0.31 (0.18–0.53)	<0.001	1.31 (0.38–4.54)	0.665	0.60 (0.33–1.12)	0.107	1.29 (0.36–4.58)	0.693	0.44 (0.28–0.68)	<0.001
Wealth quintile										
1 (lowest)	1		1		1		1		1	
2	0.89 (0.71–1.12)	0.317	0.87 (0.41–1.84)	0.714	0.91 (0.65–1.28)	0.586	1.68 (0.68–4.10)	0.258	0.85 (0.70–1.03)	0.100
3	1.03 (0.84–1.27)	0.752	1.17 (0.56–2.45)	0.675	0.93 (0.66–1.29)	0.651	1.47 (0.61–3.57)	0.389	0.94 (0.78–1.13)	0.497
4	0.76 (0.60–0.94)	0.015	0.80 (0.37–1.70)	0.553	1.08 (0.74–1.57)	0.703	1.61 (0.71–3.67)	0.256	0.83 (0.67–1.03)	0.094
5 (highest)	0.68 (0.52–0.90)	0.007	0.94 (0.41–2.16)	0.890	0.99 (0.69–1.44)	0.974	2.78 (1.16–6.70)	0.022	0.82 (0.65–1.02)	0.076
Smoking										
Non-smoker	1		1		1		1		1	
Daily smoker	1.18 (0.89–1.57)	0.237	1.11 (0.53–2.35)	0.778	0.95 (0.75–1.21)	0.674	2.24 (1.22–4.13)	0.010	0.97 (0.80–1.16)	0.714
Nondaily smoke	0.64 (0.38–1.07)	0.086	0.38 (0.05–2.81)	0.340	1.24 (0.74–2.08)	0.413	0.22 (0.04–1.15)	0.073	0.91 (0.62–1.34)	0.636
Former smoker	1.57 (1.10–2.25)	0.015	1.86 (0.79–4.40)	0.157	1.29 (0.83–1.99)	0.260	1.81 (0.86–3.79)	0.118	1.36 (1.05–1.77)	0.020
Alcohol use										
Drinker	1		1		1		1		1	
Non-drinker	1.17 (0.98–1.40)	0.088	1.09 (0.70–1.69)	0.701	1.65 (1.11–2.46)	0.014	1.24 (0.57–2.70)	0.595	1.32 (1.08–1.60)	0.006
Nutrition										
Inadequate	1		1		1		1		1	
Adequate	0.68 (0.53–0.87)	0.003	1.05 (0.65–1.69)	0.846	1.31 (0.83–2.08)	0.243	0.68 (0.42–1.10)	0.114	0.82 (0.63–1.05)	0.113
Chronic conditions										
Angina	1.12 (0.80–1.57)	0.498	2.86 (1.19–6.84)	0.019	0.89 (0.50–1.59)	0.705	1.23 (0.67–2.26)	0.495	1.07 (0.81–1.42)	0.629
Diabetes	0.93 (0.69–1.27)	0.659	2.57 (1.19–5.59)	0.017	1.46 (0.92–2.31)	0.111	1.24 (0.69–2.24)	0.472	1.24 (0.93–1.65)	0.143
Hypertension	1.22 (0.98–1.52)	0.074	0.57 (0.24–1.31)	0.184	0.79 (0.57–1.09)	0.145	2.75 (1.72–4.38)	<0.001	1.10 (0.92–1.31)	0.305
Country										
China									1	
Ghana									0.21 (0.15–0.28)	<0.001
India									1.51 (1.14–1.99)	0.004
South Africa									0.86 (0.62–1.18)	0.349

Compared with those with no schooling, respondents with higher education in China were significantly less likely to be edentulous and respondents in South Africa with primary or secondary education were significantly more likely to be edentulous, when all other variables were held constant.

In the multivariable regressions, compared with non-smokers, former smokers in China were significantly more likely to be edentulous (OR 1.57; 95 % CI 1.10–2.25), and daily smokers in South Africa were significantly more likely to be edentulous (OR 2.24; 95 % CI 1.22–4.13). In the presence of all other variables, compared with drinkers, non-drinkers in India were significantly more likely to be edentulous (OR 1.65; 95 % CI 1.11–2.46) and respondents with adequate nutrition in China were significantly less likely to be edentulous (OR 0.68; 95 % CI 0.53–0.87). In the multivariable analysis in Ghana, respondents with angina were significantly more likely to be edentulous (OR 2.86; 95 % CI 1.19–6.84), respondents with diabetes in Ghana were significantly more likely to be edentulous (OR 2.57; 95 % CI 1.19–5.59), and respondents with hypertension in South Africa were significantly more likely to be edentulous (OR 2.75; 95 % CI 1.72–4.38).

In the multivariable regression of the data set for all countries pooled, the likelihood of edentulism was higher amongst those who were older and had less education. Holding all other variables constant, compared with 50–59 year olds, respondents aged 80+ years were seven times more likely to be edentulous (OR 7.67; 95 % CI 5.93–9.91), and those with university education were 60 % less likely to be edentulous compared with those with no education (OR 0.44; 95 % CI 0.28–0.68). Compared with drinkers, non-drinkers were 30 % more likely to be edentulous when all other variables were held constant (OR 1.32; 95 % CI 1.08–1.60). Compared with the reference country China and holding all other variables constant, respondents in India were 50 % more likely to be edentulous (OR 1.51; 95 % CI 1.14–1.99) and respondents in Ghana were 80 % less likely to be edentulous (OR 0.21; 95 % CI 0.15–0.28).

## Discussion

This study of self-reported edentulism in adults aged 50 and above in four LMICs has two sets of important findings. The first is in relation to differences in the prevalence of self-reported edentulism in the two major emerging economies in Asia – China and India, and in contrast, two rapidly developing countries in the African continent – Ghana and South Africa. The second main finding relates to the distinct country-level differences in common risk factors for edentulism.

The high prevalence of edentulism in India (15.3 %) is consistent with other findings. The World Health Organization [44] estimated the prevalence of edentulism among 65–74 year olds in India at 19 % [44] and Peltzer et al. [50] reported edentulism prevalence in India at 16.3 % (95 % CI 14.3–18.4). Of the 1240 elderly Indian subjects examined in their study, Sha and Sundaram showed that 15.2 % were edentulous [46]. There has been a general trend in India to extract diseased teeth, especially among older adults and many take the view that teeth loss is a natural part of ageing [46].

Similar to the results of a previous study of oral health in SAGE countries [50] the prevalence in Ghana was only about 3 %, but three times higher in China and South Africa at 8–9 %. In younger African adults (aged 35–44 years) edentulism prevalence has been estimated at 1 % [7]. One reason for this is that many people in African countries live in rural communities where there is limited access to refined sugars, although this is changing with increasing urbanisation. However, urban to rural migration is occurring at different levels and rates in the African continent, which may also be a reason for the observed country differences in edentulism. In South Africa in 2010, only about 38 % of the population lived in rural areas compared with 42 % ten years earlier, while in Ghana about half the population lived in rural areas in 2010, compared with 58 % in 2000 [56].

The 9 % prevalence of edentulism in China in people aged 50 and over is consistent with the results of a national epidemiological survey conducted in China in 2005 in which edentulism prevalence was reported at 7 % in the 65–74 year age group and similar to a WHO estimate of 11 % [44]. China is also facing rapid rural to urban migration which, as noted above, is impacting on oral health [57]. For example about 60 % of the Chinese population lived in rural areas in 2000 compared with about 50 % in 2010 [56].

Between country differences are shown in the results of the pooled multivariable regression. After adjusting for sex, age, residence, education wealth, smoking, alcohol, nutrition, and the self-reported chronic conditions angina, diabetes and hypertension, compared with the reference country, China, respondents in India were significantly 50 % more likely to be edentulous and respondents in Ghana were significantly 80 % less likely to be edentulous. These findings are broadly consistent with WHO 2000 estimates of edentulism prevalence in 65 to 74 year olds of 11 % in China and 19 % in India [9, 44].

The association between tooth loss and older age has been widely reported in studies conducted in many countries throughout the world [6, 20, 49, 58–61]. In the univariable analyses, differences in edentulism prevalence were significant across the four age categories (50–59, 50–69, 70–79 and 80+) in China, Ghana and India, but not South Africa. However, in China and India only, the likelihood of edentulism was significantly higher in all age groups, compared with the reference group (50–59 years) after adjusting for sex, residence, education wealth, smoking, alcohol, nutrition, and the self-reported chronic conditions angina, diabetes and hypertension. In the two African countries, the association between older age and edentulism was significant only when comparing the 50–59 and 80+ year groups. When a regressor is categorised the placing of cut points can influence results [62]. In order to investigate the sensitivity of the association between age and edentulism, we re-analysed the data using three, instead of four, age categories - 50–59 years vs. 60–69 years vs. 70+ years. The age groups 70–79 and 80+ years were collapsed because of relatively small cell sizes. As with the first analysis, there were positive age gradients in each of the countries, and in Ghana and South Africa the odds were significant only for the oldest 70+ age group.

In the univariable analysis, women in China were 30 % more likely to be edentulous but the association attenuated to non-significance after adjusting for age, residence, education wealth, smoking, alcohol, nutrition and the chronic conditions angina, diabetes and hypertension. In contrast, the association between sex and edentulism was not significant in Ghana in the univariable analysis, but in the multivariable analysis, women in Ghana were significantly 60 % more likely to be edentulous. Compared with the univariable regression, the multivariable regression for Ghana showed a lower likelihood of edentulism for the 80+ age group, rural residents, and those with self-reported angina and diabetes, suggesting that these factors confounded association between sex and edentulism in the univariable model.

However, evidence of associations between sex and edentulism in older adults is mixed. Observational evidence that females are more likely to be edentulous [21, 63, 64] has been attributed to both biological and social factors. Studies have shown association between osteoporosis and oestrogen deficiency and periodontal diseases and tooth loss [65–67]. In some societies women, particularly those in higher socioeconomic groups, may be more concerned about their dental and facial appearance than men and therefore e more likely to opt for dentures when available [24, 64]. Other research shows that men tend to be more edentulous than women [68] or that the sex differences are not significant [10, 69] although this varies according to the populations studied [6]. It is important to note that differences in oral health are a function of both biological sex and gender with the later referring to behaviours resulting from the societal and cultural construction of male/female roles [23].

In China respondents in rural areas were significantly 40 % more likely to be edentulous while rural respondents in Ghana and South Africa were significantly 50 % less likely to be edentulous after adjusting for sex, age, education wealth, smoking, alcohol, nutrition, and the self-reported chronic conditions angina, diabetes and hypertension. Comparisons between urban and rural residents are complicated by the availability of and access to oral health treatment [6, 18]. In the African continent these issues are complicated by a limited oral health workforce and poor working conditions as well as logistic problems in reaching people in rural communities [7]. Dental treatments and extractions are less likely to be available in poorer rural communities. However, factors such as family and social support and informal networks can also play a role in promoting oral health care [70].

Respondents with higher levels of completed education were significantly less likely to be edentulous in China, after adjusting for sex, age, residence, wealth, smoking, alcohol, nutrition, and the self-reported chronic conditions angina, diabetes and hypertension. In the multivariable models, education to primary or secondary levels compared with no schooling, was significantly associated with edentulism in South Africa but the edentulism/education association was not significant for Ghana or India. A number of studies show that higher education is protective of edentulism in older adults [10, 20, 21]. This can be explained by the role played by education in promoting the utilization of oral health services where available.

After adjusting for sex, age, residence, education, smoking, alcohol, nutrition, and the self-reported chronic conditions angina, diabetes and hypertension, the likelihood of edentulism in the highest compared with the lowest wealth quintile was significant and 30 % less in China, and significantly almost three times higher in South Africa. Many studies in the literature report that oral health, like general health, is associated with lower socioeconomic status and wealth. However there apparent contradictory results may be due to a perception, which has been documented in African countries, that loss of teeth is an inevitable consequence of ageing. Elders are held in high regard in many traditional cultures in African countries, although this is gradually changing with increasing economic and social change [7, 45]. Another factor is that teeth extraction is seen in African cultures as being a socially acceptable solution for dental problems [10].

The patterns in the health behaviours smoking, alcohol use and nutrition are variable, possibly due to inconsistencies in self-reported responses. Adjusting for sex, age, residence, education, wealth, alcohol, nutrition, and the self-reported chronic conditions angina, diabetes and hypertension, former smokers in China were significantly 60 % more likely to be edentulous compared with non-smokers and in South Africa, daily smokers were significantly 20 % more likely to be edentulous than non-smokers. Smoking is widely cited as a risk factor for poor oral health including tooth loss [13, 71, 72]. The findings that non-drinkers in India were significantly 60 % more likely to be edentulous in the fully adjusted model is somewhat surprising given evidence of association between alcohol use and poor oral health [16, 72, 73]. However this is partly explained by the high proportion of non-drinkers (84 %) in the India study sample. In the fully adjusted models, respondents with an adequate intake of fruits and vegetables were significantly 30 % less likely to be edentulous in China, while in the other three countries the association between nutrition and edentulism was not significant. Diets rich in saturated fats and refined sugars and low in fibres and vitamins have been associated with NCDs including dental caries in a number of countries [17, 74, 75]. The result for China may reflect the traditional Chinese diet being high in fruits and vegetables, although this is now changing with increased economic development [44].

In the fully adjusted model, in Ghana, respondents with angina were significantly three times more likely to be edentulous and respondents with diabetes were significantly 50 % less likely to be edentulous. In South Africa respondents with hypertension were significantly almost three times more likely to be edentulous in the presence of sex, age, residence, education, wealth, smoking, alcohol, nutrition, angina and diabetes. There is evidence to support biological links between NCDs and oral health. Oral bacterial infections that normally precede tooth loss can influence systemic inflammatory and homeostatic factors such as C - reactive protein and leucocytes leading to vascular damage and atherosclerosis [76] and it has been suggested that having fewer teeth may affect healthier nutrient intake leading to increased risk of vascular diseases [77].

### Limitations

It was not possible to establish causation because of the analyses were cross-sectional. Although the data collection was tightly controlled, it is possible that some respondents did not answer “honestly”, particularly in relation to questions about smoking and alcohol. We also acknowledge the possibility of selection bias with different life expectancies in these four countries.

### Strengths

The WHO-SAGE data are collected in a highly consistent manner. To our knowledge, this is the first study of its kind to use standardised data and definitions to specifically investigate common risk factor patterns of edentulousness in these four LMICs. The analysis conducted separately for each country provides insights not possible with only a pooled multi-country analysis. The results provide a platform for further work to help build an evidence-base to inform the development of context-specific policies on oral health in LMICs.

## Conclusions

The study calls for strengthening of the CRFA for oral and other NCDs [78]. The focus should be on the common determinants of health, community participation, partnerships with other sectors, healthy public policies and reducing health inequalities and inequities to achieve improvements in oral and overall health.

## Abbreviations

CI, confidence interval; CRFA, common risk factor approach; DALYs, Disability adjusted life years; HICs, high-income countries; LMICs, low-and middle-income countries; NCDs, non-communicable diseases; OR, odds ratio; SAGE, Study on global AGEing and adult health; WHO, World Health Organization
